# Korean medicine inpatient care: trends and influencing factors

**DOI:** 10.3389/fmed.2025.1611609

**Published:** 2025-09-15

**Authors:** Boram Lee, Changsop Yang, Mi Hong Yim

**Affiliations:** ^1^KM Science Research Division, Korea Institute of Oriental Medicine, Daejeon, Republic of Korea; ^2^Digital Health Research Division, Korea Institute of Oriental Medicine, Daejeon, Republic of Korea

**Keywords:** traditional medicine, Korean medicine, hospitalization, Korea Health Panel Survey, health care utilization

## Abstract

**Background:**

Despite the high preference and effectiveness of Korean medicine inpatient care (KMIC), including herbal medicine and acupuncture, South Korea’s national health insurance coverage for Korean medicine remains limited, accounting for only 4% of the total national health insurance expenditures. We aimed to analyze the status and related factors of KMIC for better integration and resource allocation.

**Methods:**

This cross-sectional study included 1,362 inpatient care users from 2022 Korea Health Panel Survey data. To examine factors associated with the use of KMIC, weighted multivariable logistic regression analyses were conducted using survey sampling weights based on Andersen’s behavioral model. To summarize patient experiences, satisfaction, and KMIC or conventional medicine inpatient care (CMIC) use, weighted estimates were calculated using survey sampling weights.

**Results:**

Female gender (adjusted odds ratio [95% confidence interval], 3.26 [1.18, 9.01]) and regular physical activity (2.15 [1.13, 4.09]) were associated with a greater likelihood of KMIC use. The likelihood of KMIC use was also greater in individuals aged 45–59 years than in those aged 19–44 years (3.11 [1.24, 7.79]), and in residents of Gwangju/Jeolla/Jeju than in those of Seoul/Incheon/Gyeonggi/Gangwon (7.35 [3.35, 16.13]). Moreover, individuals with poor/very poor perceived health status showed a greater likelihood of KMIC use than those with very good/good perceived health status (3.06 [1.05, 8.94]). Musculoskeletal disorders were the primary diagnosis for both KMIC and CMIC use. Patient satisfaction was rated as “very satisfied” or “satisfied” in 70%–82% of cases of KMIC use, except in the category of hospitalization costs.

**Conclusion:**

Korean medicine inpatient care (KMIC) use was more common among females, individuals with poor subjective health status, and individuals engaging in regular physical activity. Patients primarily used KMIC for the treatment of accidents and musculoskeletal disorders and generally reported high levels of satisfaction.

## 1 Introduction

Traditional medicine (TM) plays a significant role in healthcare systems worldwide, with utilization rates varying across regions (1). In Africa, up to 80% of the population is estimated to rely on TM for their primary healthcare needs (2). In developed countries, the use of specific TM modalities, such as acupuncture and homeopathy, is also notable. A systematic review reported that the 12-month prevalence of visits to acupuncturists ranged from 0.2 to 7.5%, while that of visits to homeopaths ranged from 0.2 to 2.9% (3).

In South Korea, Korean TM, such as acupuncture, moxibustion, and herbal medicine, is widely utilized and holds a significant place in the healthcare system. Approximately 25.4% of South Korean individuals use Korean medicine treatment every year (4), and patient satisfaction with Korean medicine services is notably high (5). Korean medicine inpatient care (KMIC) is also actively utilized, particularly for conditions like stroke rehabilitation and musculoskeletal disorders (6, 7). A retrospective cohort study examined the long-term outcomes of ischemic stroke patients who received adjuvant Korean medicine treatments and suggested a potential improvement in survival rates (7). Another study found that an integrative rehabilitation program combining Korean medicine and physical therapy significantly improved pain, disability, and shoulder range of motion after arthroscopic rotator cuff repair, causing no adverse events or retears (6). These findings underscore the prominent role of Korean medicine in South Korea’s healthcare system, particularly in inpatient care.

Several countries have integrated TM services into their national health insurance systems (1, 8). In particular, South Korea’s national health insurance has covered Korean medicine services, including acupuncture and herbal preparations, since 1987, marking the first instance of a TM system being insured nationwide (8). Despite its inclusion, the coverage for Korean medicine remains limited compared to that for conventional medicine in South Korea. The medical expenditure for Korean medicine under South Korea’s national health insurance is only 4% of the total (4). Limited financial support from the national health insurance increases out-of-pocket expenses and restricts access to care (8). This financial strain may reduce the utilization of Korean medicine, even though it is associated with high patient satisfaction and therapeutic effectiveness (6, 7, 9). Therefore, understanding utilization patterns and key factors influencing Korean medicine is essential for optimizing resource allocation and improving its integration into the national health insurance system (10). Studies have assessed the status and related factors of Korean medicine treatment use based on healthcare big data analysis (10–13). However, these studies have analyzed the outpatient use of Korean medicine or focused on specific diseases. To the best of our knowledge, no study has analyzed the patterns and determinants of overall KMIC. Therefore, this study aimed to assess the status and related factors of KMIC by analyzing nationally representative healthcare big data.

## 2 Materials and methods

### 2.1 Data source and study participants

**FIGURE 1:**
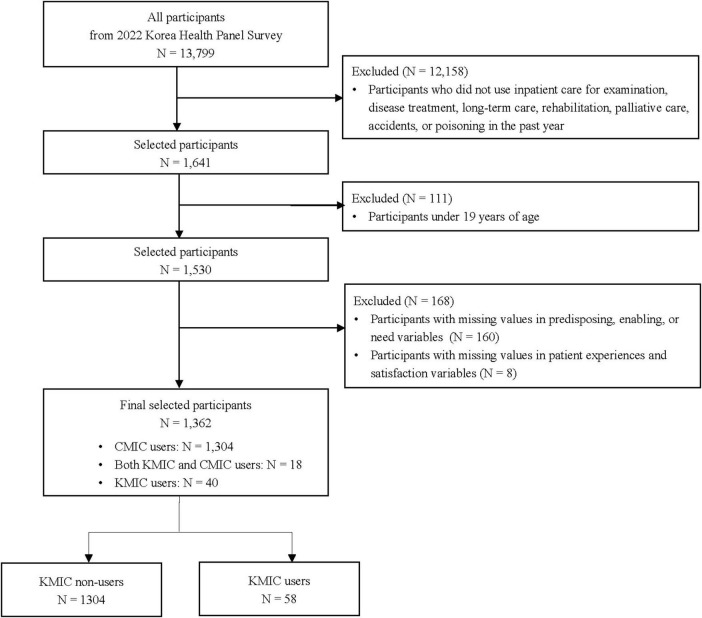
Flowchart of participant selection process. CMIC, conventional medicine inpatient care; KMIC, Korean medicine inpatient care.

This cross-sectional study was conducted using data from the 2022 Korea Health Panel Survey (KHPS), conducted jointly by the Korea Institute for Health and Social Affairs and the National Health Insurance Service. The KHPS is a nationwide panel survey that revisits the same households each year, compiling detailed data on healthcare use and spending in Korea. Although the KHPS provides multi-year data, this study focused on the most recent available year, 2022, to capture current trends and influencing factors of KMIC use. Recognizing the KHPS’s complex sampling design, we applied complex sample analytic methods that explicitly incorporate these features, thereby preserving survey representativeness and improving the precision of our statistical estimates. It examines healthcare service utilization, medical expenditures, and their influencing factors, including demographic and socioeconomic characteristics, pharmaceutical and medical service expenses, chronic disease management, health-related perceptions and behaviors, and private health insurance. It is implemented through face-to-face interviews, where trained interviewers visit households, collect responses, and record them using computer-assisted personal interviewing techniques. Data from the KHPS are provided in a de-identified format to prevent the identification of individuals and ensure compliance with the Personal Information Protection Act and the Statistics Act. Access to KHPS data is open to the public; however, it requires signing a data use agreement and submitting it to the designated administrator^1^. An exemption for this study was granted by the Institutional Review Board (IRB) at the Korea Institute of Oriental Medicine because of its reliance on the secondary analysis of de-identified data (IRB No. I-2504/004-002). A total of 13,799 individuals participated in the 2022 KHPS. Of these, 12,158 participants who had not utilized inpatient care in the past year for examination, disease treatment, long-term care, rehabilitation, palliative care, accidents, or poisoning were excluded from the study. Moreover, 111 participants under 19 years of age, 160 participants with missing values in predisposing, enabling, or need variables, and eight participants with missing values in patient experiences and satisfaction variables were excluded. Finally, 1,362 participants were included in this study: 1,304 conventional medicine inpatient care (CMIC) users, 18 users of both KMIC and CMIC, and 40 KMIC users (Figure 1).

### 2.2 Outcome and other variables

The outcome was KMIC use for examination, treatment, long-term care, rehabilitation, palliative care, accidents, or poisoning in the past year. The KMIC use group consisted of individuals who used only KMIC or both KMIC and CMIC, while the KMIC non-use group consisted of individuals who used only CMIC. To improve the accuracy of healthcare utilization data, participants were instructed to maintain a health expense log documenting their healthcare usage over the past year, including medical receipts and year-end tax records. Based on these records, healthcare utilization data for individuals were collected through self-reported responses, which were verified by interviewers through a review of the health expense log, medical receipts, and year-end tax records.

Demographic and socioeconomic characteristics, chronic diseases, and health-related perceptions and behaviors were analyzed as factors associated with KMIC use. These variables were categorized as predisposing, enabling, and need factors according to Andersen’s behavioral model of health services use (14–17) and analyzed to evaluate their influence on KMIC use. Predisposing factors refer to demographic and social characteristics that precede healthcare utilization, enabling factors refer to resources or conditions that support or limit healthcare utilization, and need factors refer to perceived or actual health conditions that drive healthcare utilization (18–21). Predisposing factors included sex (male or female), age (19–44, 45–59, 60–74, or ≥ 75 years), region (Seoul/Incheon/Gyeonggi/Gangwon, Daejeon/Sejong/Chungcheong, Busan/Daegu/Ulsan/Gyeongsang, or Gwangju/Jeolla/Jeju), education level (elementary school or below, middle/high school, or college or above), and marital status (married/living together or widowed/divorced/separated/never married). Enabling factors included the number of household members (one, two, three, or four or more), household income (first quartile [lowest], second quartile, third quartile, or fourth quartile [highest]), employment status (unpaid family worker/unemployed, employed, or self-employed), and private health insurance (no or yes). Need factors included perceived health status (very good/good, fair, or poor/very poor), perceived stress (barely, low, or high/very high), depressed mood (no or yes), anxious mood (no or yes), regular physical activities (no or yes), alcohol use (none, monthly or less, or two or more times a month), cigarette use (no or yes), body mass index (BMI) (< 23, 23–24.9, or ≥ 25), hypertension (no or yes), diabetes mellitus (no or yes), cardiovascular and cerebrovascular diseases (no or yes), and malignant neoplasm (no or yes).

Several variables were examined to explore patient experiences, satisfaction, and KMIC or CMIC use. To investigate inpatient experience and satisfaction, various parameters were analyzed, including the primary reason for choosing the healthcare institutions, the individual with the maximum influence on admission and treatment decisions, the receipt of collaborative treatment from other departments during hospital stay, the primary caregiver during hospital stay, the receipt of unnecessary treatments or tests during hospital stay, and satisfaction across seven dimensions. To examine inpatient care use, the main reason for hospitalization, primary diagnosis for hospital admissions, treatments received during hospital stay, healthcare costs, and length of hospital stay were summarized.

### 2.3 Statistical analysis

For all statistical analyses, survey sampling weights were applied to account for the complex sampling design of the KHPS, producing estimates generalizable to the entire Korean population. Data analyses were performed using R version 4.4.1 (R Foundation for Statistical Computing, Vienna, Austria) for data preprocessing and the complex samples procedure of IBM SPSS Statistics for Windows version 29.0 (IBM Corp., Armonk, NY, United States) for weighted estimates and statistical modeling. All statistical tests employed two-sided approaches with the significance level set at 0.05.

To compare general characteristics between KMIC users and non-users, Pearson’s chi-squared tests, adjusted with the second-order Rao–Scott correction for complex survey designs, were used for categorical variables (22). The results were presented as unweighted frequencies and weighted column proportions. Weighted logistic regression analyses were conducted to identify the association between KMIC use and predisposing, enabling, and need factors. In the unadjusted analysis, a univariable logistic regression model was used to identify the association between each variable of predisposing, enabling, and need factors and KMIC use. In the adjusted analysis, a multivariable logistic regression model was used to identify the association between multiple variables of these factors and KMIC use. The results were reported as unadjusted and adjusted odds ratios (uORs and aORs, respectively) with 95% confidence intervals (CIs) for the respective analyses. Model adequacy for the fully adjusted weighted logistic regression was evaluated with the Nagelkerke’s and McFadden’s pseudo-R^2^ statistics. Multicollinearity was evaluated using generalized variance inflation factors (GVIFs) obtained from an unweighted logistic regression, as existing survey modules cannot yet compute factor-level GVIFs and sampling weights do not modify the correlation matrix among categorical predictors.

To summarize patient experiences, satisfaction, and KMIC or CMIC use, weighted estimates were calculated using survey sampling weights. The results were summarized as unweighted frequencies and weighted column proportions for categorical variables and weighted means and standard errors for continuous variables. For the analysis of patient experiences, satisfaction, and inpatient care use, KMIC use was defined as either the exclusive use of KMIC or the combined use of KMIC and CMIC. Similarly, CMIC use was defined as either the exclusive use of CMIC or the combined use of KMIC and CMIC. As the related variables were collected on a case-by-case basis and KMIC use and CMIC use were not mutually exclusive, statistical tests could not be performed. A subgroup analysis was conducted among patients hospitalized due to accidents or poisoning to investigate their characteristics, determinants of KMIC use, as well as their care experiences, satisfaction, and treatment details.

## 3 Results

### 3.1 General characteristics between KMIC users and non-users

A total of 1,362 individuals were included in this study; these included 1,304 KMIC non-users and 58 KMIC users. The proportion of females was higher among KMIC users (71.84%) than among KMIC non-users (56.24%). The highest proportion of individuals aged 45–49 years was observed among KMIC users (56.18%), while the highest proportion of individuals aged 60–74 years was noted among KMIC non-users (32.01%). Moreover, residents of Gwangju/Jeolla/Jeju accounted for the highest proportion of KMIC users (41.72%), while residents of Seoul/Incheon/Gyeonggi/Gangwon accounted for the highest proportion of KMIC non-users (48.67%). The proportion of KMIC users enrolled in private health insurance (93.78%) was higher than that of KMIC non-users (76.33%) (Table 1). In subgroup analysis among accidents or poisoning inpatients, 241 individuals were identified, comprising 29 patients who used KMIC and 212 patients who exclusively used CMIC. The proportion of participants aged 45–59 years (KMIC users, 53.82%; vs KMIC non-users 25.91%), residing in Gwangju/Jeolla/Jeju (35.27% vs 12.74%), with highest household income (46.39% vs. 28.90%), and engaging in regular exercise (75.93% vs 36.88%) was higher among KMIC users than among KMIC non-users (Supplementary Table 1).

**TABLE 1:** General characteristics between users and non-users of Korean medicine inpatient care.

Variables	Total	KMIC non-use	KMIC use	*P-*value
Number of participants	1362	1304	58	
**Predisposing factors**				
Sex				0.045
Men Women	569 (43.2)	552 (43.76)	17 (28.16)	
	793 (56.8)	752 (56.24)	41 (71.84)	
Age				0.002
19–44 45–59 60–74 75 or older	144 (20.72)	136 (20.96)	8 (14.25)	
	254 (31.01)	231 (30.08)	23 (56.18)	
	587 (31.7)	564 (32.01)	23 (23.37)	
	377 (16.56)	373 (16.95)	4 (6.2)	
Region				<0.001
Seoul/Incheon/Gyeonggi/Gangwon Daejeon/Sejong/Chungcheong Busan/Daegu/Ulsan/Gyeongsang Gwangju/Jeolla/Jeju	354 (48.19)	343 (48.67)	11 (35.24)	
	246 (11.64)	240 (11.83)	6 (6.59)	
	417 (26.12)	409 (26.48)	8 (16.45)	
	345 (14.06)	312 (13.03)	33 (41.72)	
Education level				0.241
Elementary school or below Middle/high school College or above	481 (21.67)	472 (22.05)	9 (11.44)	
	630 (47.58)	597 (47.27)	33 (55.77)	
	251 (30.76)	235 (30.68)	16 (32.8)	
Marital status				0.465
Married/living together Widowed/divorced/separated/never married	395 (32.35)	382 (32.55)	13 (26.9)	
	967 (67.65)	922 (67.45)	45 (73.1)	
**Enabling factors**				
Number of household members				0.515
1 2 3 4 or more	248 (17.16)	242 (17.36)	6 (11.95)	
	710 (35.03)	682 (34.68)	28 (44.69)	
	188 (22.31)	177 (22.52)	11 (16.54)	
	216 (25.49)	203 (25.44)	13 (26.82)	
Household income				0.105
1st quartile (lowest) 2nd quartile 3rd quartile	370 (19.46)	365 (19.93)	5 (6.64)	
	369 (21.18)	356 (21.27)	13 (18.85)	
	297 (22.96)	280 (22.57)	17 (33.45)	
4th quartile (highest)	326 (36.4)	303 (36.23)	23 (41.06)	
Employment status				0.055
Unpaid family worker/unemployed Employed Self-employed	709 (43.19)	692 (43.85)	17 (25.31)	
	467 (44.94)	436 (44.35)	31 (60.84)	
	186 (11.87)	176 (11.8)	10 (13.84)	
Private health insurance	915 (76.94)	859 (76.33)	56 (93.48)	0.036
**Need factors**				
Perceived health status				0.978
Very good/good Fair Poor/very poor	343 (27.58)	324 (27.54)	19 (28.65)	
	531 (44.08)	511 (44.14)	20 (42.54)	
	488 (28.33)	469 (28.32)	19 (28.81)	
Perceived stress				0.951
Barely Low High/very high	299 (19.86)	287 (19.85)	12 (20.14)	
	677 (49.38)	649 (49.31)	28 (51.34)	
	386 (30.76)	368 (30.84)	18 (28.52)	
Depressed mood	115 (9.36)	110 (9.43)	5 (7.73)	0.688
Anxiety mood	73 (6.27)	71 (6.32)	2 (4.78)	0.690
Regular physical activities	661 (47.78)	627 (47.22)	34 (62.89)	0.054
Alcohol use				0.416
None Monthly or less 2 or more times a month	705 (42.57)	681 (42.65)	24 (40.51)	
	278 (22.08)	266 (22.32)	12 (15.7)	
	379 (35.35)	357 (35.04)	22 (43.79)	
Cigarette use	195 (16.57)	185 (16.52)	10 (17.97)	0.808
BMI				0.744
<23 23–24.9 ≥25	557 (41.33)	535 (41.19)	22 (44.85)	
	366 (26.09)	347 (26.02)	19 (28.15)	
	439 (32.58)	422 (32.79)	17 (27)	
Hypertension	634 (34.24)	613 (34.53)	21 (26.47)	0.277
Diabetes mellitus	277 (15.45)	270 (15.61)	7 (11.19)	0.403
Cardiovascular and cerebrovascular diseases	222 (11.82)	213 (11.65)	9 (16.5)	0.387
Malignant neoplasm	143 (8.7)	137 (8.58)	6 (12.13)	0.462

BMI, body mass index; KMIC, Korean medicine inpatient care. The values show unweighted frequencies (weighted column proportions) for categorical variables. *P-*values were calculated using Pearson’s chi-squared tests, adjusted with the second-order Rao–Scott correction for complex survey designs.

### 3.2 Factors associated with KMIC use

In the unadjusted analysis identifying each variable of predisposing, enabling, and need factors associated with KMIC use, significant associations were observed between KMIC use and the following variables: sex, age, region, household income, and employment status. Females were more likely to use KMIC than males (uOR [95% CI], 1.99 [1.004, 3.924]). Individuals aged 45 × 59 years also had a higher tendency to use KMIC than those aged 19 × 44 years (2.75 [1.10, 6.89]). Moreover, residents of Gwangju/Jeolla/Jeju had a greater likelihood of KMIC use than those of Seoul/Incheon/Gyeonggi/Gangwon (4.42 [2.03, 9.61]). Compared to the lowest income quartile, higher household income quartiles were associated with a higher tendency to use KMIC (third quartile, 4.45 [1.37, 14.40]; fourth quartile (highest), 3.4 [1.12, 10.36]). Additionally, employed workers were more likely to use KMIC than unpaid family workers or unemployed individuals (2.38 [1.10, 5.12]).

In the fully adjusted analysis examining the associations between combined variables of predisposing, enabling, and need factors and KMIC use, a significant association was noted between KMIC use and sex, age, region, perceived health status, and regular physical activities. Females were associated with a greater likelihood of KMIC use than males (aOR [95% CI], 3.26 [1.18, 9.01]). Individuals aged 45–59 years were also more likely to use KMIC than those aged 19–44 years (3.11 [1.24, 7.79]). Moreover, residents of Gwangju/Jeolla/Jeju were more likely to use KMIC than those of Seoul/Incheon/Gyeonggi/Gangwon (7.35 [3.35, 16.13]). Additionally, individuals with poor/very poor perceived health status exhibited a greater likelihood of KMIC use than those with very good/good perceived health status (3.06 [1.05, 8.94]). Regular physical activity was also associated with increased KMIC use (2.15 [1.13, 4.09]). The fully adjusted model achieved a Nagelkerke’s pseudo-R^2^ of 0.234 and a McFadden’s pseudo-R^2^ of 0.208, reflecting acceptable explanatory capacity. All degree-adjusted (scaled) GVIF values were below 1.39 (range 1.05–1.39), far below the conventional threshold of concern, confirming that multicollinearity was negligible (23) (Table 2, Supplementary Figure 1, and Supplementary Table 2). In subgroup analysis among accidents or poisoning inpatients, individuals living in Gwangju/Jeolla/Jeju compared with Seoul/Incheon/Gyeonggi/Gangwon, those who perceived their health status as fair versus very good/good, those reporting depressed mood, those with regular physical activities, and individuals with cardiovascular or cerebrovascular disease were more likely to use KMIC (Supplementary Table 1).

**TABLE 2:** Association between the use of Korean medicine inpatient care and predisposing, enabling, and need factors.

Variables	Unadjusted analysis		Adjusted analysis	
	uOR (95% CI)	*P*-value	aOR (95% CI)	*P*-value
**Predisposing factors**				
Sex				
Men	1 [Reference]		1 [Reference]	
Women	1.99 (1.004, 3.924)	0.049	3.26 (1.18, 9.01)	0.023
Age				
19–44	1 [Reference]		1 [Reference]	
45–59	2.75 (1.1, 6.89)	0.031	3.11 (1.24, 7.79)	0.015
60–74	1.07 (0.43, 2.71)	0.879	0.81 (0.22, 2.95)	0.752
75 or older	0.54 (0.14, 2.04)	0.362	0.94 (0.11, 8.31)	0.958
Region				
Seoul/Incheon/Gyeonggi/Gangwon	1 [Reference]		1 [Reference]	
Daejeon/Sejong/Chungcheong	0.77 (0.25, 2.34)	0.644	0.71 (0.22, 2.25)	0.56
Busan/Daegu/Ulsan/Gyeongsang	0.86 (0.31, 2.37)	0.767	1.31 (0.49, 3.52)	0.596
Gwangju/Jeolla/Jeju	4.42 (2.03, 9.61)	<0.001	7.35 (3.35, 16.13)	<0.001
Education level				
Elementary school or below	1 [Reference]		1 [Reference]	
Middle/high school	2.27 (0.92, 5.6)	0.074	1.32 (0.45, 3.88)	0.608
College or above	2.06 (0.79, 5.4)	0.141	1.83 (0.41, 8.19)	0.427
Marital status				
Married/living together	1 [Reference]		1 [Reference]	
Widowed/divorced/separated/never married	0.76 (0.37, 1.58)	0.466	1.17 (0.39, 3.55)	0.777
**Enabling factors**				
Number of household members				
1	1 [Reference]		1 [Reference]	
2	1.87 (0.64, 5.46)	0.251	2.65 (0.78, 9.07)	0.119
3	1.07 (0.32, 3.57)	0.917	0.9 (0.22, 3.64)	0.877
4 or more	1.53 (0.48, 4.85)	0.469	0.76 (0.17, 3.41)	0.719
Household income				
1st quartile (lowest)	1 [Reference]		1 [Reference]	
2nd quartile	2.66 (0.8, 8.84)	0.11	1.73 (0.44, 6.82)	0.436
3rd quartile	4.45 (1.37, 14.4)	0.013	2.78 (0.66, 11.71)	0.163
4th quartile (highest)	3.4 (1.12, 10.36)	0.031	2.44 (0.57, 10.43)	0.229
Employment status				
Unpaid family worker/unemployed	1 [Reference]		1 [Reference]	
Employed	2.38 (1.1, 5.12)	0.027	2.43 (0.9, 6.62)	0.081
Self-employed	2.03 (0.76, 5.42)	0.157	2.12 (0.62, 7.23)	0.228
Private health insurance				
No	1 [Reference]		1 [Reference]	
Yes	4.44 (0.97, 20.44)	0.055	2.69 (0.27, 26.52)	0.397
**Need factors**				
Perceived health status				
Very good/good	1 [Reference]		1 [Reference]	
Fair	0.93 (0.44, 1.97)	0.842	1.81 (0.79, 4.16)	0.163
Poor/very poor	0.98 (0.44, 2.17)	0.957	3.06 (1.05, 8.94)	0.041
Perceived stress				
Barely	1 [Reference]		1 [Reference]	
Low	1.03 (0.44, 2.4)	0.953	0.6 (0.25, 1.47)	0.266
High/very high	0.91 (0.37, 2.25)	0.84	0.71 (0.23, 2.16)	0.545
Depressed mood				
No	1 [Reference]		1 [Reference]	
Yes	0.8 (0.28, 2.33)	0.688	1.34 (0.42, 4.24)	0.622
Anxiety mood				
No	1 [Reference]		1 [Reference]	
Yes	0.74 (0.17, 3.22)	0.691	0.48 (0.08, 2.8)	0.416
Regular physical activities				
No	1 [Reference]		1 [Reference]	
Yes	1.89 (0.98, 3.67)	0.058	2.15 (1.13, 4.09)	0.02
Alcohol use				
None	1 [Reference]		1 [Reference]	
Monthly or less	0.74 (0.32, 1.7)	0.479	0.6 (0.24, 1.48)	0.265
2 or more times a month	1.32 (0.63, 2.74)	0.464	1.4 (0.59, 3.31)	0.448
Cigarette use				
No	1 [Reference]		1 [Reference]	
Yes	1.11 (0.49, 2.52)	0.808	1.58 (0.54, 4.58)	0.403
BMI				
<23	1 [Reference]		1 [Reference]	
23–24.9	0.99 (0.45, 2.18)	0.987	1.02 (0.46, 2.27)	0.956
≥25	0.76 (0.35, 1.65)	0.482	0.8 (0.31, 2.02)	0.634
Hypertension				
No	1 [Reference]		1 [Reference]	
Yes	0.68 (0.34, 1.36)	0.28	0.74 (0.31, 1.8)	0.511
Diabetes mellitus				
No	1 [Reference]		1 [Reference]	
Yes	0.68 (0.28, 1.68)	0.406	0.63 (0.21, 1.88)	0.402
Cardiovascular and cerebrovascular diseases				
No	1 [Reference]		1 [Reference]	
Yes	1.5 (0.6, 3.77)	0.39	3.4 (0.95, 12.24)	0.061
Malignant neoplasm				
No	1 [Reference]		1 [Reference]	
Yes	1.47 (0.52, 4.16)	0.465	1.72 (0.51, 5.78)	0.378

aOR, adjusted odds ratio; BMI, body mass index; CI, confidence interval; uOR, unadjusted odds ratio. The values represent unadjusted and adjusted odds ratios with 95% confidence intervals for the unadjusted and adjusted analyses, respectively. In the unadjusted analysis, a univariable logistic regression model was used to identify the association between each variable of predisposing, enabling, and need factors and the use of Korean medicine inpatient care. In the adjusted analysis, a multivariable logistic regression model was used to assess the association between multiple variables of these factors and the use of Korean medicine inpatient care. In all statistical analyses, survey sampling weights were applied to account for the complex survey design.

### 3.3 Patient experiences, satisfaction, and inpatient care use

Regarding patient experiences and satisfaction with inpatient care, among the 1,362 inpatients included in this study, a total of 2,111 inpatient care episodes were recorded; these included 2,017 cases of CMIC and 94 cases of KMIC. The primary reason for choosing the healthcare institutions, representing the highest proportion, was the superior medical staff, accounting for 68.74% of cases among KMIC users and 48.62% of cases among CMIC users. Assessment of the individual who had the greatest influence on admission and treatment decisions revealed that the most influential person was the patient for KMIC use (67.86%) and the doctor or physician for CMIC use (57.66%). In total, 68.84% of KMIC users received collaborative treatment with CMIC; however, only 0.28% of CMIC users received collaborative treatment with KMIC. The proportion of inpatient care without a caregiver was 93.77% for KMIC use and 54.64% for CMIC use. Regarding the receipt of unnecessary treatments or tests during hospital stay, the proportion of cases where the respondents answered “strongly disagree” was 56.2% for KMIC use and 16.13% for CMIC use. Regarding satisfaction with the use of inpatient care, the proportion of cases where the respondents answered “very satisfied/satisfied” was as follows for KMIC and CMIC use: 86.47 and 74.74% for the choice of medical staff, 89.22 and 79.14% for the adequacy and accuracy of medical staff explanations, 92.49 and 82.25% for the attitude of hospital staff and medical personnel, 87.05 and 74.17% for the length of hospital stay, 91.05 and 78.01% for the appropriateness and adequacy of medical treatment, and 89.41 and 79.12% for hospital facilities and equipment, respectively. Regarding hospitalization costs, the proportion of cases with no out-of-pocket expenses was 13.18% for KMIC use and 2.56% for CMIC use. Additionally, 67.55% of KMIC users responded as “neutral” in terms of hospitalization costs, while 52.19% of CMIC users responded as “very satisfied/satisfied” (Table 3).

**TABLE 3:** Summary of patient experiences and satisfaction with the use of inpatient care.

Variables	CMIC use	KMIC use
Valid cases	2017	94
**Primary reason for choosing the healthcare institutions**		
Superior medical staff	1021 (48.62)	55 (68.74)
Advanced equipment and facilities	139 (8.1)	6 (7.25)
Proximity to home	228 (10.85)	15 (11.44)
Regular healthcare institutions	522 (25.92)	11 (7.63)
Other	107 (6.51)	7 (4.94)
**Individual with the most influence on admission and treatment decisions**		
Doctor/physician	1181 (57.66)	36 (25.85)
Patient himself/herself	655 (36.29)	53 (67.86)
Family member	173 (5.84)	2 (0.69)
Other	8 (0.21)	3 (5.59)
**Receipt of collaborative treatment from other departments during hospital stay**		
No	2010 (99.72)	41 (31.16)
Yes	7 (0.28)	53 (68.84)
**Primary caregiver during hospital stay**		
Family member	863 (39)	11 (5.36)
Paid caregiver	121 (5.68)	1 (0.87)
No caregiver	1015 (54.64)	82 (93.77)
Other	18 (0.69)	0 (0)
**Receipt of unnecessary treatment or tests during hospital stay**		
Strongly agree/somewhat agree	99 (4.54)	4 (2.77)
Disagree	1558 (79.33)	50 (41.04)
Strongly disagree	360 (16.13)	40 (56.2)
**Satisfaction with choice of medical staff**		
Very satisfied/satisfied	1490 (74.74)	70 (86.47)
Neutral	490 (23.86)	23 (12.83)
Dissatisfied/very dissatisfied	37 (1.4)	1 (0.7)
**Satisfaction with adequacy and accuracy of medical staff explanations**		
Very satisfied/satisfied	1584 (79.14)	77 (89.22)
Neutral	400 (18.84)	16 (9.96)
Dissatisfied/very dissatisfied	33 (2.01)	1 (0.83)
**Satisfaction with attitude of hospital staff and medical personnel**		
Very satisfied/satisfied	1642 (82.25)	82 (92.49)
Neutral	351 (16.14)	11 (6.68)
Dissatisfied/very dissatisfied	24 (1.6)	1 (0.83)
**Satisfaction with length of hospital stay**		
Very satisfied/satisfied	1523 (74.17)	76 (87.05)
Neutral	436 (22.96)	16 (11.37)
Dissatisfied/very dissatisfied	58 (2.87)	2 (1.57)
**Satisfaction with appropriateness and adequacy of medical treatment**		
Very satisfied/satisfied	1563 (78.01)	79 (91.05)
Neutral	423 (20.59)	15 (8.95)
Dissatisfied/very dissatisfied	31 (1.4)	0 (0)
**Satisfaction with hospital facilities and equipment**		
Very satisfied/satisfied	1589 (79.12)	78 (89.41)
Neutral	406 (19.35)	15 (10.26)
Dissatisfied/very dissatisfied	22 (1.53)	1 (0.33)
**Satisfaction with hospitalization costs**		
Very satisfied/satisfied	1018 (52.19)	22 (16.84)
Neutral	747 (34.41)	52 (67.55)
Dissatisfied/very dissatisfied	205 (10.84)	4 (2.44)
No payment	47 (2.56)	16 (13.18)

CMIC, Conventional medicine inpatient care; KMIC, Korean medicine inpatient care. The values indicate unweighted frequencies (weighted column proportions) for categorical variables. Weighted column proportions were calculated using survey sampling weights to account for the complex survey design.

The use of inpatient care, including primary diagnosis, treatments, costs, and length of hospital stay, was also summarized. The proportions of cases with examination and disease treatment, accidents or poisoning, and long-term care, rehabilitation, and palliative care as the main reasons for hospitalization were 69.52%, 26.92%, and 3.56% for KMIC use, respectively, and 86.06%, 12.49%, and 1.46% for CMIC use, respectively. Regarding the primary diagnosis for hospital admissions, the most common primary diagnosis for KMIC use was accidents or poisoning (26.92%), followed by joint disorders of the shoulder, pelvis, or spine (13.75%), arthritis (1.8%), and spinal disc disorders (1.27%). In contrast, the most common primary diagnosis for CMIC use was musculoskeletal disorders (19.67%), followed by malignant neoplasms (15.34%), accidents or poisoning (12.49%), and ophthalmic diseases (11.21%). The most frequently used treatment during hospital stay was physical therapy (97.57%), followed by acupuncture (95.23%), herbal decoction (75.15%), and pharmacopuncture (55.2%) for KMIC use. For CMIC use, the most frequently used treatment was surgery and procedures (54.86%), followed by non-surgical treatments (39.39%) and diagnostic tests (5.75%). The average out-of-pocket cost per case and length of hospital stay per case were 958,573 Korean Won (KRW) and 8.76 days for KMIC use, respectively, and 1,426,395 KRW and 7.79 days for CMIC use, respectively (Table 4).

**TABLE 4:** Summary of the use of inpatient care: reasons for admission, primary diagnosis, treatments, costs, and length of hospital stay.

Type of inpatient care	Variables	*N* (%)
**Conventional medicine inpatient care use**	Valid cases	2017
	**Main reason for hospitalization**	
	Examination and disease treatment	1718 (86.06)
	Accident or poisoning	260 (12.49)
	Long-term care, rehabilitation, and palliative care	39 (1.46)
	**Primary diagnosis for hospital admissions**	
	Musculoskeletal disorders	414 (19.67)
	Malignant neoplasm	304 (15.34)
	Accident or poisoning	260 (12.49)
	Ophthalmic diseases	241 (11.21)
	Gastrointestinal diseases	90 (5.72)
	Obstetric and gynecological conditions	46 (3.24)
	Ischemic heart diseases	65 (2.55)
	Stroke	55 (2.4)
	Renal and urologic diseases	56 (2.33)
	Neuropsychiatric disorders	50 (2.31)
	Diabetes mellitus	22 (1.23)
	Respiratory diseases	35 (1.1)
	Liver diseases	19 (0.77)
	Hypertension	6 (0.25)
	Other	354 (19.39)
	**Treatments received during hospital stay**	
	Surgery and procedures	1057 (54.86)
	Non-surgical treatments (e.g., medication, physical/rehabilitation therapy, blood transfusion, chemotherapy)	857 (39.39)
	Diagnostic tests	103 (5.75)
	**Healthcare costs**	
	Inpatient out-of-pocket costs (KRW/case)	1,426,395 ± 66,320
	**Day of health care uses**	
	Length of hospital stay (days/case)	7.79 ± 0.3
**Korean medicine inpatient care use**	Valid cases	94
	**Main reason for hospitalization**	
	Examination and disease treatment	56 (69.52)
	Accident or poisoning	31 (26.92)
	Long-term care, rehabilitation, and palliative care	7 (3.56)
	**Primary diagnosis for hospital admissions**	
	Accident or poisoning	31 (26.92)
	Other joint disorders (shoulder, pelvis, spine, etc.)	21 (13.75)
	Arthritis	5 (1.8)
	Spinal disc disorders (cervical, lumbar, etc.)	2 (1.27)
	Other	35 (56.26)
	**Treatments received during hospital stay**	
	Physical therapy	90 (97.57)†
	Acupuncture	88 (95.23)†
	Herbal decoction	60 (75.15)†
	Pharmacopuncture	34 (55.2)†
	Chuna	33 (52.97)†
	Moxibustion	27 (23.99)†
	Cupping therapy	35 (22.44)†
	General herbal medicine preparations (such as granule and pill)	23 (20.29)†
	Manual therapy	24 (14.42)†
	Expensive herbal medicine preparations (such as Gongjindan)	1 (0.96)†
	**Healthcare costs**	
	Inpatient out-of-pocket costs (KRW/case)	958,573 ± 90,749
	**Day of health care uses**	
	Length of hospital stay (days/case)	8.76 ± 0.68

KRW, Korean Won. The values indicate unweighted frequencies (weighted column proportions) for categorical variables. However, as the selection of treatment items for Korean medicine inpatient care allowed for multiple responses, ^†^represents the proportion of cases that received the specific treatment relative to the total number of cases. For continuous variables, the values are presented as weighted means ± standard errors. The values were calculated using survey sampling weights to account for the complex survey design.

Within the subgroup analysis of inpatients hospitalized due to accidents or poisoning, 31 KMIC cases and 260 CMIC cases were identified. Regarding the primary reason for selecting a healthcare institution, 18.91% of KMIC users cited advanced equipment and facilities, compared to 5.05% of CMIC users. In terms of patient satisfaction with the inpatient care experience—excluding the item related to hospitalization costs—the proportion of “Very satisfied/Satisfied” responses among KMIC users ranged from 75.43 to 82.15%, whereas among CMIC users, the corresponding proportions ranged from 63.81 to 77.84%. Regarding services received during hospitalization, surgery and procedures accounted for the largest proportion (57.31%) among CMIC users. In contrast, among KMIC users, physical therapy (100%) and acupuncture (97.78%) were the most commonly provided treatments. The average out-of-pocket cost per case was 163,680 KRW for KMIC and 1,308,782 KRW for CMIC. The average length of stay was 10.83 days for KMIC users and 11.99 days for CMIC users (Supplementary Table 3).

## 4 Discussion

This study is the first to analyze trends and related factors of KMIC use based on data from the KHPS, nationally representative sample data in Korea. A total of 1,362 participants were included in the analysis. Although studies have been conducted on the factors and characteristics influencing the use of Korean medicine in healthcare, they differ from this study in that they mostly targeted outpatients or analyzed them without any distinction between outpatient and inpatient status. Moreover, they focused on patients with specific diseases (10–13).

When analyzing the general characteristics of KMIC users, significant differences were noted compared to non-users only in the following predisposing and enabling factors: sex, age, region, and private health insurance. KMIC users comprised a higher proportion of females, individuals aged 45–59 years, residents of Gwangju/Jeolla/Jeju, and those with private health insurance. The likelihood of KMIC use was higher among females than males; individuals aged 45–59 years compared to those aged 19–44 years; residents of Gwangju/Jeolla/Jeju compared to those living in Seoul/Incheon/Gyeonggi/Gangwon; individuals with poor or very poor perceived health status compared to those with very good or good perceived health status; and individuals who engaged in regular physical activity.

Our findings regarding predisposing factors are consistent with those of a previous study that analyzed the National Health Insurance Service–National Sample Cohort between 2002 and 2013 (10). That study similarly found that females and individuals in their 40s and 50s were more likely to use Korean medicine treatment (10). In terms of enabling factors, while high-income groups were found to use Korean medicine treatment more than low-income groups in that study (10), our study showed no significant difference in usage across household income levels. However, it is important to note that the previous study did not focus specifically on KMIC, unlike our study (10). In addition, compared to the total number of medical institutions, the Gwangju region has the highest ratio of Korean medicine hospitals available for hospitalization (24). Among all regions, the Gwangju/Jeolla/Jeju area had the highest number of Korean medicine hospitals per 100,000 people (24). Hence, accessibility to Korean medicine hospitals may be a factor that influences the frequency of KMIC use by residents.

Regarding the need factor, the finding that individuals with poor perceived health status were more likely to use KMIC than those with good perceived health status is also consistent with a previous finding that overweight and obese female adolescents with poor subjective health status used herbal medicine, a representative Korean medicine treatment, more often (12). This may be because the prevalence of chronic diseases was found to be more than twofold higher in individuals with poor self-rated health status than in those with good self-rated health status. Moreover, Korean medicine treatments, such as herbal medicine and acupuncture, are actively used to manage chronic diseases (25–27). Individuals who engaged in regular physical activity were also more likely to use KMIC, indicating that individuals who engage in regular physical activity may experience musculoskeletal problems due to injury or overuse during exercise and may utilize KMIC because of the known effects of Korean medicine treatment on musculoskeletal disorders (28, 29).

Regarding patient experience with inpatient care, the reason for choosing healthcare institutions was the superior medical staff in both KMIC and CMIC. However, the individual who played the most important role in admission and treatment decisions was the medical staff for CMIC use and the patient for KMIC use, confirming that patient self-determination plays a greater role in KMIC use. In contrast to the very low rate of receiving collaborative treatment from other departments during hospitalization among CMIC users, a significant number of KMIC users received collaborative treatment from other departments. This may be because Korean medicine, which is based on holistic management, promotes collaborative treatment with various specialties to provide optimal treatment by considering the patient’s overall health condition. It also integrates conventional medical diagnosis and treatment with the practice of Korean medicine. KMIC users generally rated satisfaction with the choice of medical staff, adequacy and accuracy of medical staff explanations, attitudes of hospital staff and medical personnel, length of hospital stay, appropriateness and adequacy of medical treatment, and hospital facilities and equipment as “very satisfied” or “satisfied.” Similarly, in a survey of 237 traffic injury patients treated with Korean medicine, 75.1% of the patients were satisfied with their treatments, and 85.2% experienced symptom improvement, further supporting the high satisfaction levels associated with KMIC (9). However, in terms of satisfaction with hospitalization costs, the highest percentage of KMIC users responded as “neutral,” whereas among CMIC users, the highest percentage reported being “very satisfied/satisfied.” This may be partially explained by the relatively low national health insurance coverage for Korean medicine, which accounts for only 4% of total national health insurance medical expenditures (4), thereby placing a higher perceived burden on patients. Interestingly, in our study, the out-of-pocket cost per hospitalization was higher for CMIC than for KMIC. One plausible explanation is that KMIC use was more commonly associated with hospitalizations due to accidents or poisoning, particularly traffic accidents, for which most medical costs are covered by automobile insurance in South Korea. This is consistent with our finding that the actual no-payment rate was significant among KMIC users. Nevertheless, the discrepancy between lower actual costs and lower satisfaction with those costs among KMIC users may reflect factors beyond monetary burden. These could include perceived coverage gaps, limitations in reimbursed services, or a lower perceived value of care due to differences in insurance policy design between KMIC and CMIC. Patients may feel that certain Korean medicine treatments are insufficiently covered or undervalued within the current system, which could contribute to lower satisfaction, even when personal expenditures are minimal.

For CMIC use, the most common primary diagnosis for hospital admission was musculoskeletal disorders, followed by malignant neoplasm. For KMIC use, the most common primary diagnosis was accidents or poisoning, including traffic accidents, followed by joint disorders of the shoulder, pelvis, or spine, arthritis, and spinal disc disorders, with musculoskeletal disorders being common. Moreover, for KMIC use, the most common Korean medicine treatments received during hospital stay included physical therapy, acupuncture, herbal decoction, pharmacopuncture, and Chuna manual therapy. These results regarding the primary diagnosis and main treatment contents for Korean medicine use are consistent with previous findings (1, 10).

In the subgroup analysis of patients hospitalized due to accidents or poisoning, KMIC use was more likely among those living in Gwangju/Jeolla/Jeju regions compared to those in Seoul/Incheon/Gyeonggi/Gangwon, those with fair perceived health status relative to very good/good status, those experiencing depressed mood, those engaging in regular physical activity, and those with cardiovascular or cerebrovascular diseases. Although previous studies have analyzed medical records of patients hospitalized in Korean medicine hospitals due to traffic accidents (30–33), this is the first study to examine determinants of KMIC use based on a nationwide panel survey incorporating a more comprehensive set of variables that cannot be captured through medical record review. Furthermore, our study is the first to analyze the experiences, satisfaction, and treatment details of patients who used KMIC following traffic accidents. However, our analysis included not only traffic accident patients but also those hospitalized due to other types of accidents or poisoning. In addition, the relatively small number of KMIC users (*n* = 29) limits the generalizability of these findings and results in broad confidence intervals.

This study has several limitations. First, as the KHPS relies on self-reported data regarding health status, healthcare use, and expenditures, there is a potential for recall and response bias that may affect the accuracy of the results. Second, psychological variables such as depressive mood, anxiety, and perceived stress were measured using non-standardized, categorical self-report items. The lack of validated psychometric tools reduces measurement reliability; however, such simplified indicators are commonly used in large-scale national surveys to broadly capture health-related perceptions. Third, the KHPS collects pre-determined variables, and not all clinically relevant information may have been captured. For example, the multiple-choice options for diseases or reasons for KMIC use were primarily focused on musculoskeletal and neurological conditions. This may have led to underreporting of KMIC utilization for other common indications, such as respiratory or digestive diseases, potentially biasing the observed treatment patterns. Consequently, the findings may underestimate the broader scope of KMIC utilization, and caution is warranted when generalizing these patterns and diagnoses to all inpatient KMIC users. Fourth, although we adjusted for a wide range of demographic, enabling, and behavioral factors, residual confounding remains possible. Important clinical and cultural variables—such as illness severity, comorbidities, and cultural attitudes toward Korean medicine—were not available in the KHPS dataset and may have influenced both healthcare utilization and satisfaction. Fifth, the potential for self-selection bias cannot be ruled out. Individuals with a preference for Korean medicine or specific socioeconomic characteristics may have been more likely to choose KMIC over CMIC, which may have affected the associations observed. Sixth, although the KHPS is a nationally representative survey, the findings may not be generalizable to populations outside South Korea or to outpatient care settings. Seventh, in the analysis of patient experiences, satisfaction, and inpatient care use, because inpatient use was summarized at the event level, a single individual could contribute admissions to both the KMIC and CMIC sets and could appear multiple times within a set. These overlapping and non-independent observations precluded formal statistical comparisons of total events. This limitation should be taken into account when interpreting event-level findings. Finally, as this study analyzed only the 2022 KHPS data collected through a cross-sectional survey, it is difficult to establish clear causal relationships between the identified variables. In addition, the limited sample size of KMIC users (*n* = 58) may have resulted in imprecise estimates, as reflected in the wide confidence intervals. Therefore, the conclusions should be interpreted with caution and generalized carefully.

Nevertheless, to our knowledge, this is the first study to comprehensively examine KMIC using KHPS data, providing timely insights into traditional medicine use and patient experiences in a real-world, nationally representative context. Given that Korean medicine accounts for only 4% of national health insurance expenditures, the limited financial support may restrict access and increase out-of-pocket costs, potentially discouraging utilization despite high levels of patient satisfaction and reported effectiveness (4). By analyzing recent national health panel data, this study fills an important gap left by previous research that focused primarily on outpatient care or specific diseases. Our findings highlight key factors associated with KMIC use and offer valuable insights for optimizing resource allocation and improving the integration of Korean medicine into the broader national health insurance system. To translate these findings into effective policy, a more nuanced and targeted approach is needed that addresses barriers such as limited reimbursement coverage for Korean medicine services, variability in practitioner training, and the need for consistent quality control across institutions. Addressing these structural and administrative challenges could improve the accessibility, equity, and efficiency of KMIC delivery. Overall, our findings provide timely and practical evidence to inform health policy efforts aimed at advancing the integration and long-term sustainability of Korean medicine within the broader healthcare system.

## 5 Conclusion

Analysis of the 2022 KHPS data revealed that KMIC use was more prevalent among females and residents of Gwangju/Jeolla/Jeju. Moreover, individuals with poor/very poor perceived health status and those engaging in regular physical activity were more likely to use KMIC. Patients primarily made their own admission and treatment decisions, and their overall satisfaction with KMIC use was high. The common primary diagnosis for KMIC use was accidents and musculoskeletal disorders.

## Data Availability

The original contributions presented in this study are included in this article/Supplementary material, further inquiries can be directed to the corresponding author.

## Footnotes

1
https://www.khp.re.kr:444/
