# Supplementary material for: Korean medicine inpatient care: trends and influencing factors
Source: Front Med (Lausanne). 2025 Sep 15;12:1611609. doi: 10.3389/fmed.2025.1611609 (PMC12477256; doi:10.3389/fmed.2025.1611609)
Supplement: Supplementary file 1 [file Data_Sheet_1.docx]

**Supplementary Figure S1. Adjusted odds ratio for factors associated with the use of Korean medicine inpatient care**

#
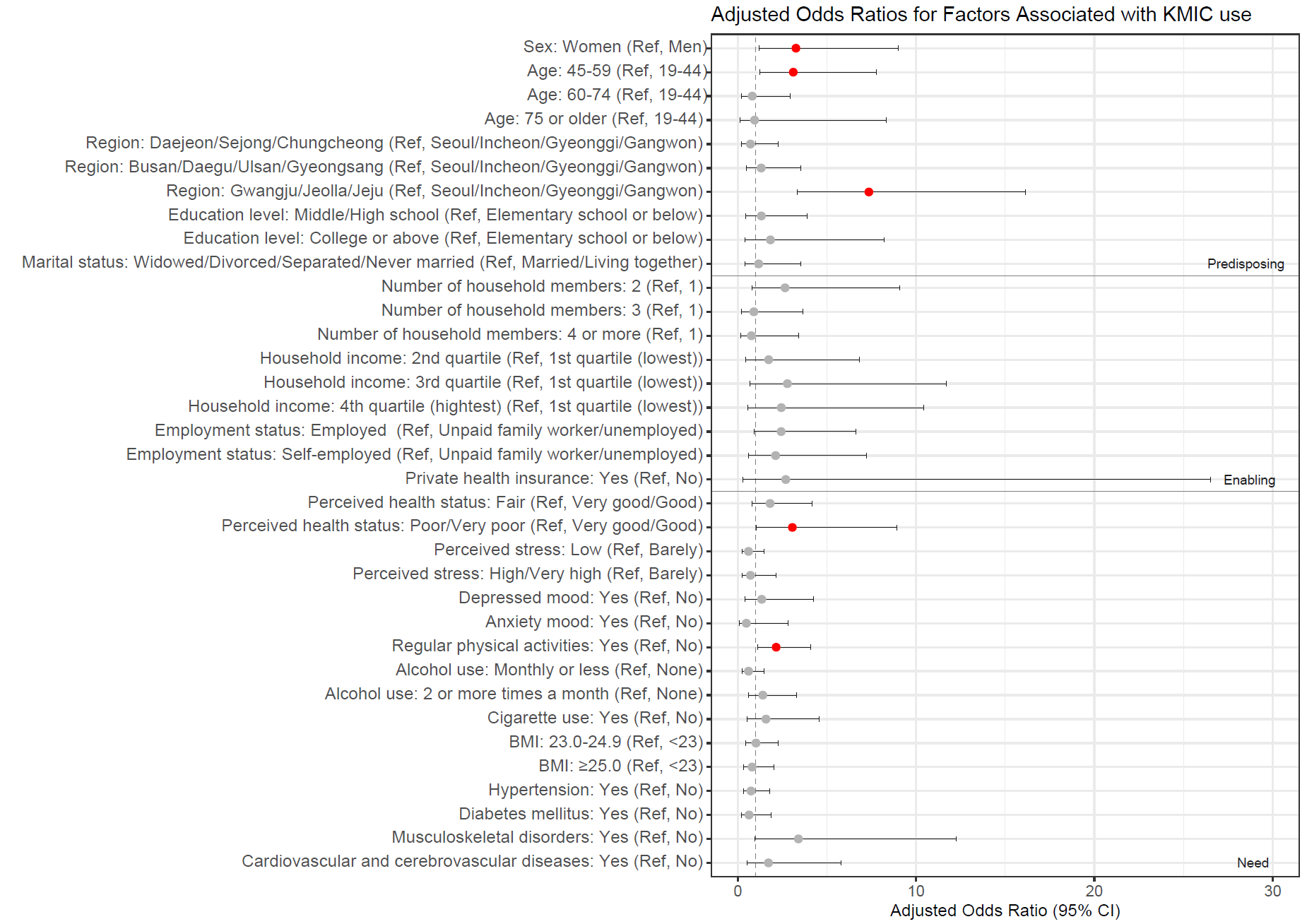


# BMI, body mass index; KMIC, Korean medicine inpatient care.

**Supplementary Table S1. Characteristics and determinants of KMIC use among accidents or poisoning inpatients**

| **Variables** | **Comparison by KMIC use** | | | **Adjusted analysis** | |
| --- | --- | --- | --- | --- | --- |
|  | **KMIC non-use** | **KMIC use** | ***P value*** | **aOR (95% CI)** | ***P* value** |
| Number of participants | 212 | 29 |  |  |  |
| **Predisposing factors** |  |  |  |  |  |
| Sex |  |  | 0.214 |  |  |
| Men | 84 (47.45) | 10 (32.73) |  | 1 [Reference] |  |
| Women | 128 (52.55) | 19 (67.27) |  | 0.69 (0.1, 4.62) | 0.702 |
| Age |  |  | 0.027 |  |  |
| 19-44 | 22 (25.46) | 7 (22.92) |  | 1 [Reference] |  |
| 45–59 | 37 (25.91) | 11 (53.82) |  | 1.64 (0.2, 13.51) | 0.646 |
| 60–74 | 95 (29.14) | 9 (19.34) |  | 0.22 (0.02, 2.83) | 0.243 |
| 75 or older | 58 (19.49) | 2 (3.92) |  | 0.24 (0.01, 7.85) | 0.417 |
| Region |  |  | 0.014 |  |  |
| Seoul/Incheon/Gyeonggi/Gangwon | 44 (43.56) | 7 (44.59) |  | 1 [Reference] |  |
| Daejeon/Sejong/Chungcheong | 49 (13.74) | 3 (5.06) |  | 0.26 (0.02, 2.68) | 0.255 |
| Busan/Daegu/Ulsan/Gyeongsang | 66 (29.95) | 5 (15.08) |  | 1.37 (0.23, 8.26) | 0.727 |
| Gwangju/Jeolla/Jeju | 53 (12.74) | 14 (35.27) |  | 27.77 (4.61, 167.41) | <0.001 |
| Education level |  |  | 0.37 |  |  |
| Elementary school or below | 80 (23.12) | 4 (9.88) |  | 1 [Reference] |  |
| Middle/High school | 98 (49.12) | 15 (54.92) |  | 0.84 (0.12, 5.66) | 0.858 |
| College or above | 34 (27.76) | 10 (35.2) |  | 0.36 (0.02, 8.33) | 0.523 |
| Marital status |  |  | 0.35 |  |  |
| Married/Living together | 150 (63.46) | 22 (73.98) |  | 1 [Reference] |  |
| Widowed/Divorced/Separated/Never married | 62 (36.54) | 7 (26.02) |  | 4.5 (0.76, 26.66) | 0.097 |
| **Enabling factors** |  |  |  |  |  |
| Number of household members |  |  | 0.344 |  |  |
| 1 | 39 (20.01) | 3 (9.4) |  | 1 [Reference] |  |
| 2 | 109 (34.86) | 12 (40.74) |  | 8.49 (0.8, 90.33) | 0.076 |
| 3 | 33 (26.44) | 5 (17.05) |  | 1.28 (0.08, 21.9) | 0.862 |
| 4 or more | 31 (18.69) | 9 (32.81) |  | 5.35 (0.49, 58.3) | 0.168 |
| Household income |  |  | 0.003 |  |  |
| 1st quartile (lowest) | 61 (20.27) | 1 (0.51) |  | 1 [Reference] |  |
| 2nd quartile | 53 (23.35) | 3 (5.36) |  | 181.01 (0.14, 227399.74) | 0.152 |
| 3rd quartile | 51 (27.48) | 11 (47.75) |  | 1075.02 (0.57, 2023899.77) | 0.069 |
| 4th quartile (highest) | 47 (28.9) | 14 (46.39) |  | 1343.93 (0.34, 5375850.91) | 0.088 |
| Employment status |  |  | 0.79 |  |  |
| Unpaid family worker/unemployed | 109 (37.6) | 10 (32.82) |  | 1 [Reference] |  |
| Employed | 78 (51.55) | 15 (58.88) |  | 0.38 (0.07, 1.95) | 0.243 |
| Self-employed | 25 (10.84) | 4 (8.3) |  | 0.04 (0, 0.76) | 0.033 |
| Private health insurance |  |  | 0.181 |  |  |
| No | 74 (24.69) | 1 (7.92) |  | 1 [Reference] |  |
| Yes | 138 (75.31) | 28 (92.08) |  | 4.67 (0.74, 29.39) | 0.1 |
| **Need Factors** |  |  |  |  |  |
| Perceived health status |  |  | 0.327 |  |  |
| Very good/Good | 67 (36.46) | 11 (34.24) |  | 1 [Reference] |  |
| Fair | 79 (39.33) | 14 (54.52) |  | 22.88 (1.79, 291.84) | 0.016 |
| Poor/Very poor | 66 (24.21) | 4 (11.25) |  | 1.76 (0.14, 21.66) | 0.657 |
| Perceived stress |  |  | 0.702 |  |  |
| Barely | 55 (26.58) | 5 (17.44) |  | 1 [Reference] |  |
| Low | 102 (45.46) | 14 (50.78) |  | 1.6 (0.32, 8.02) | 0.566 |
| High/Very high | 55 (27.97) | 10 (31.78) |  | 5.1 (0.69, 37.58) | 0.109 |
| Depressed mood |  |  | 0.414 |  |  |
| No | 193 (92.65) | 25 (87.81) |  | 1 [Reference] |  |
| Yes | 19 (7.35) | 4 (12.19) |  | 13.79 (1.6, 118.83) | 0.017 |
| Anxiety mood |  |  | 0.41 |  |  |
| No | 199 (95.54) | 27 (91.54) |  | 1 [Reference] |  |
| Yes | 13 (4.46) | 2 (8.46) |  | 0.22 (0.01, 4.14) | 0.313 |
| Regular physical activities |  |  | 0.001 |  |  |
| No | 129 (63.12) | 9 (24.07) |  | 1 [Reference] |  |
| Yes | 83 (36.88) | 20 (75.93) |  | 25.34 (3.75, 171.37) | 0.001 |
| Alcohol use |  |  | 0.77 |  |  |
| None | 105 (38.8) | 12 (38.98) |  | 1 [Reference] |  |
| Monthly or less | 42 (16.29) | 5 (10.87) |  | 0.28 (0.02, 3.73) | 0.334 |
| 2 or more times a month | 65 (44.91) | 12 (50.15) |  | 0.97 (0.2, 4.71) | 0.967 |
| Cigarette use |  |  | 0.729 |  |  |
| No | 180 (78.57) | 23 (81.84) |  | 1 [Reference] |  |
| Yes | 32 (21.43) | 6 (18.16) |  | 0.69 (0.06, 7.72) | 0.759 |
| BMI |  |  | 0.519 |  |  |
| <23 | 83 (39.21) | 12 (47.58) |  | 1 [Reference] |  |
| 23-24.9 | 60 (25.75) | 11 (30.72) |  | 5.26 (0.71, 38.82) | 0.103 |
| ≥25 | 69 (35.04) | 6 (21.7) |  | 1.12 (0.13, 9.41) | 0.919 |
| Hypertension |  |  | 0.121 |  |  |
| No | 119 (61.05) | 21 (78.81) |  | 1 [Reference] |  |
| Yes | 93 (38.95) | 8 (21.19) |  | 0.36 (0.04, 3.58) | 0.381 |
| Diabetes mellitus |  |  | 0.387 |  |  |
| No | 170 (84.15) | 27 (91.36) | 0.387 | 1 [Reference] |  |
| Yes | 42 (15.85) | 2 (8.64) |  | 0.29 (0.02, 3.35) | 0.319 |
| Cardiovascular and cerebrovascular diseases |  |  | 0.062 |  |  |
| No | 190 (90.22) | 23 (75.01) |  | 1 [Reference] |  |
| Yes | 22 (9.78) | 6 (24.99) |  | 10.3 (1.83, 57.82) | 0.008 |
| Malignant neoplasm |  |  |  |  |  |
| No | 198 (94.17) | 27 (94.72) | 0.919 | 1 [Reference] |  |
| Yes | 14 (5.83) | 2 (5.28) |  | 0.91 (0.11, 7.78) | 0.93 |

BMI, body mass index; KMIC, Korean medicine inpatient care; aOR, adjusted odds ratio; CI, confidence interval; uOR, unadjusted odds ratio.

When comparing KMIC use among accidents or poisoning inpatients, the values show unweighted frequencies (weighted column proportions) for categorical variables. P values were calculated using Pearson’s chi-squared tests, adjusted with the second-order Rao–Scott correction for complex survey designs. For adjusted analysis, the values represent adjusted odds ratios with 95% confidence intervals. A multivariable logistic regression model was used to assess the association between multiple variables of these factors and the use of Korean medicine inpatient care. In all statistical analyses, survey sampling weights were applied to account for the complex survey design.

**Supplementary Table S2. Scaled GVIFs for all categorical covariates** **(degree‑adjusted)**

| Variables | Degree of freedom | Scaled GVIF |
| --- | --- | --- |
| Sex | 1 | 1.28 |
| Age | 3 | 1.25 |
| Region | 3 | 1.05 |
| Education level | 2 | 1.22 |
| Marital status | 1 | 1.39 |
| Number of household members | 3 | 1.22 |
| Household income | 3 | 1.10 |
| Employment status | 2 | 1.12 |
| Private health insurance | 1 | 1.11 |
| Perceived health status | 2 | 1.16 |
| Perceived stress | 2 | 1.07 |
| Depressed mood | 1 | 1.21 |
| Anxiety mood | 1 | 1.18 |
| Regular physical activities | 1 | 1.08 |
| Alcohol use | 2 | 1.11 |
| Cigarette use | 1 | 1.21 |
| BMI | 2 | 1.05 |
| Hypertension | 1 | 1.20 |
| Diabetes mellitus | 1 | 1.10 |
| Cardiovascular and cerebrovascular diseases | 1 | 1.14 |
| Malignant neoplasm | 1 | 1.07 |

BMI, body mass index.

**Supplementary Table S3. Patient experience, satisfaction, and care details among accidents or poisoning inpatients**

| **Variables** | **CMIC use** | **KMIC use** |
| --- | --- | --- |
| Valid cases | 260 | 31 |
| Primary reason for choosing the healthcare institutions |  |  |
| Superior medical staff | 99 (33.19) | 10 (32.63) |
| Advanced equipment and facilities | 12 (5.05) | 3 (18.91) |
| Proximity to home | 62 (23.65) | 11 (31.96) |
| Regular healthcare institutions | 64 (22.49) | 4 (8.19) |
| Other | 23 (15.63) | 3 (8.31) |
| Individual with the most influence on admission and treatment decisions |  |  |
| Doctor/Physician | 138 (56.93) | 14 (42.97) |
| Patient Himself/Herself | 91 (32.99) | 14 (36.26) |
| Family Member | 27 (9.42) | 0 (0) |
| Other | 4 (0.67) | 3 (20.77) |
| Receipt of collaborative treatment from other departments during hospital stay |  |  |
| No | 258 (99.6) | 20 (68.23) |
| Yes | 2 (0.4) | 11 (31.77) |
| Primary caregiver during hospital stay |  |  |
| Family member | 99 (38.66) | 5 (11.54) |
| Paid caregiver | 32 (15.77) | 0 (0) |
| No caregiver | 126 (44.84) | 26 (88.46) |
| Other | 3 (0.73) | 0 (0) |
| Receipt of unnecessary treatment or tests during hospital stay |  |  |
| Strongly agree/Somewhat agree | 8 (2.99) | 2 (5.09) |
| Disagree | 211 (84.27) | 23 (82.01) |
| Strongly disagree | 41 (12.74) | 6 (12.89) |
| Satisfaction with choice of medical staff |  |  |
| Very satisfied/Satisfied | 175 (63.81) | 21 (75.43) |
| Neutral | 81 (34.93) | 10 (24.57) |
| Dissatisfied/Very dissatisfied | 4 (1.25) | 0 (0) |
| Satisfaction with adequacy and accuracy of medical staff explanations |  |  |
| Very satisfied/Satisfied | 200 (77.69) | 23 (80.93) |
| Neutral | 56 (19.45) | 8 (19.07) |
| Dissatisfied/Very dissatisfied | 4 (2.86) | 0 (0) |
| Satisfaction with attitude of hospital staff and medical personnel |  |  |
| Very satisfied/Satisfied | 205 (77.84) | 24 (82.15) |
| Neutral | 52 (20.41) | 7 (17.85) |
| Dissatisfied/Very dissatisfied | 3 (1.75) | 0 (0) |
| Satisfaction with length of hospital stay |  |  |
| Very satisfied/Satisfied | 181 (67.05) | 23 (75.47) |
| Neutral | 70 (28.49) | 8 (24.53) |
| Dissatisfied/Very dissatisfied | 9 (4.45) | 0 (0) |
| Satisfaction with appropriateness and adequacy of medical treatment |  |  |
| Very satisfied/Satisfied | 187 (70.27) | 23 (80.28) |
| Neutral | 70 (27.43) | 8 (19.72) |
| Dissatisfied/Very dissatisfied | 3 (2.29) | 0 (0) |
| Satisfaction with hospital facilities and equipment |  |  |
| Very satisfied/Satisfied | 191 (70.55) | 22 (76.66) |
| Neutral | 67 (27.49) | 8 (22.11) |
| Dissatisfied/Very dissatisfied | 2 (1.96) | 1 (1.23) |
| Satisfaction with hospitalization costs |  |  |
| Very satisfied/Satisfied | 114 (45.28) | 8 (22.95) |
| Neutral | 100 (32.21) | 6 (27.01) |
| Dissatisfied/Very dissatisfied | 25 (13.13) | 2 (3.88) |
| No payment | 21 (9.37) | 15 (46.16) |
| Treatments received during hospital stay |  |  |
| Surgery and procedures | 128 (57.31) |  |
| Non-surgical treatments (e.g., medication, physical/rehabilitation therapy, blood transfusion, chemotherapy) | 127 (38.91) |  |
| Diagnostic tests | 5 (3.78) |  |
| Physical therapy |  | 31 (100) † |
| Acupuncture |  | 30 (96.78) † |
| Moxibustion |  | 11 (44.92) † |
| Herbal decoction |  | 12 (38.72) † |
| General herbal medicine preparations (such as granule and pill) |  | 9 (33.96) † |
| Cupping therapy |  | 12 (31.86) † |
| Manual therapy |  | 9 (21.62) † |
| Pharmacopuncture |  | 4 (8.94) † |
| Chuna |  | 1 (4.21) † |
| Expensive herbal medicine preparations (such as Gongjindan) |  | 1 (3.58) † |
| Healthcare costs |  |  |
| Inpatient out-of-pocket costs (KRW/case) | 1,308,782± 134,399 | 163,680 ± 45,776 |
| Day of health care uses |  |  |
| Length of hospital stay (days/case) | 11.99± 0.83 | 10.83 ± 1.35 |

CMIC, Conventional medicine inpatient care; KMIC, Korean medicine inpatient care; KRW, Korean Won.

The values indicate unweighted frequencies (weighted column proportions) for categorical variables. However, as the selection of treatment items for Korean medicine inpatient care allowed for multiple responses, † represents the proportion of cases that received the specific treatment relative to the total number of cases. For continuous variables, the values are presented as weighted means ± standard errors. The values were calculated using survey sampling weights to account for the complex survey design.
